# Systematic analysis of adverse reactions associated with dantrolene treatment: From clinical features to molecular mechanisms

**DOI:** 10.1097/MD.0000000000046304

**Published:** 2026-05-12

**Authors:** Haohui Chen, Xulin Zhang, Dongxu Chen, Xiaoqin Jiang, Chao Yu

**Affiliations:** a Department of Anesthesiology, West China Second University Hospital, Sichuan University, Chengdu, People’s Republic of China; b Key Laboratory of Birth Deficits and Related Diseases of Women and Children, Sichuan University, Ministry of Education, Chengdu, People’s Republic of China; c West China School of Medicine, Sichuan University, Chengdu, People’s Republic of China.

**Keywords:** adverse reaction, dantrolene, malignant hyperthermia, pharmacovigilance databases

## Abstract

Dantrolene, 1st synthesized in 1967, is widely used for treating malignant hyperthermia (MH), neuroleptic malignant syndrome, and spasticity. However, comprehensive analysis of its adverse effects and underlying mechanisms remains limited. This study aims to analyze dantrolene-associated adverse events (AEs) using pharmacovigilance databases, with particular focus on subgroup analysis comparing MH versus non-MH patients, and investigate their underlying molecular mechanisms. We analyzed AEs from 3 pharmacovigilance databases (U.S. Food and Drug Administration Adverse Event Reporting System [2004–2024], Japanese Adverse Drug Event Report [2004–2023], Canada Vigilance Adverse Reaction Online Database [1991–2019]) and conducted differential gene expression analysis using GEO datasets (GSE184769, GSE227229). Signal detection employed disproportionality analysis using reporting odds ratio (ROR), proportional reporting ratio, Bayesian confidence propagation neural network, and Empirical Bayesian Geometric Mean indices. Analysis revealed significant respiratory and musculoskeletal AEs. Respiratory failure was consistently reported across databases (ROR: 29.33–46.29), while musculoskeletal complications included rhabdomyolysis (ROR: 14.05) and compartment syndrome (ROR: 80.52). MH patients showed increased risks of muscular weakness (ROR: 19.00), respiratory failure (ROR: 5.48), and pulmonary edema (ROR: 22.18). Molecular analysis identified IL6 and ALB as key mediators of respiratory effects, while Adgrl1 and Adgrl2 emerged as crucial regulators of muscle function. Our study quantified differential dantrolene susceptibility, revealing significantly higher AE risks in MH versus non-MH patients: muscular weakness (ROR: 19.00), respiratory failure (ROR: 5.48), and pulmonary edema (ROR: 22.18). Molecular analysis demonstrated that mutant ryanodine receptor 1 channels amplify IL6/ALB-mediated respiratory effects and disrupt Adgrl1/2-regulated muscle function, establishing the mechanistic basis for MH patient vulnerability and supporting MH-specific dosing strategies.

## 1. Introduction

Dantrolene was 1st synthesized in 1967 and has established itself as a crucial therapeutic agent with multiple clinical applications. Initially developed and widely used as an oral antispasticity drug alongside diazepam,^[1,2]^ dantrolene’s therapeutic scope expanded significantly following the demonstration of its efficacy in preventing and treating malignant hyperthermia (MH) in pigs in 1975,^[3]^ which led to its approval for MH treatment in the United States in 1979. Additionally, dantrolene is utilized in the treatment of neuroleptic malignant syndrome (NMS), where its ability to inhibit skeletal muscle contraction effectively alleviates NMS-related symptoms.^[4,5]^ In vivo, dantrolene undergoes micrometabolism in hepatic cells, primarily being converted to 5-hydroxydantrolene, a compound with skeletal muscle relaxant properties.^[6]^ Both dantrolene and its metabolites are predominantly excreted via urine and bile.^[7]^

MH is a life-threatening pharmacogenetic disorder triggered by exposure to volatile anesthetics (e.g. halothane, isoflurane) and depolarizing muscle relaxants (e.g. succinylcholine), leading to a hypermetabolic response in skeletal muscle. Current research indicates that mutations in the ryanodine receptor 1 (RYR1) and calcium channel, voltage-dependent, L type, alpha 1S subunit (CACNA1S) genes are closely associated with the pathogenesis of MH.^[8]^ The symptoms of MH include elevated end-tidal carbon dioxide levels, rapid and severe hyperthermia, muscle rigidity, apnea, tachycardia, hyperkalemia, and rhabdomyolysis. During general anesthesia, the incidence of MH ranges from 1:62,000 to 1:84,000. The presence of muscle rigidity or spasms during anesthesia induction may indicate an impending MH crisis.^[9]^ As the lack of recognition of MH, the mortality rate can be as high as 64% without effective treatment.^[10]^ MH has become a major risk factor threatening the intraoperative life safety of patients. Dantrolene is the 1st choice in the treatment of MH. Early diagnosis of MH and timely use of dantrolene are key to limit symptom progression and reduce mortality. A systematic review by Larach et al assessed the relationship between dantrolene use and MH-related morbidity and mortality, showing that the incidence of complications increased with each 10-minute delay in dantrolene use, and reached 100% with a 50-minute delay.^[11]^ By interfering with calcium ion release from the skeletal muscle endoplasmic reticulum, dantrolene reduces cytoplasmic calcium concentrations. This mechanism inhibits excessive skeletal muscle contraction while maintaining normal neuromuscular action potential patterns. Dantrolene is also very effective in the treatment of many non-MH diseases, such as antipsychotic malignant syndrome, MDMA toxicity, etc.^[8,12]^

The effectiveness and wide application of dantrolene in clinical practice make its adverse reactions sometimes ignored by clinicians. The adverse reactions related to dantrolene reported by DAILYMED were updated in 2006. In a US study of 1044 patients who took dantrolene and were followed for a long time, 19 (1.8%) had significant liver damage and 6 (0.6%) had significant jaundice. Also, there have been cases of dantrolene-induced pharyngeal spasm.^[13]^ This could have serious consequences. However, current studies on dantrolene’s adverse reactions have several limitations. First, while individual adverse reactions have been reported, there is limited comprehensive analysis of the full spectrum of dantrolene-associated adverse events (AEs) using large-scale pharmacovigilance databases. Second, existing literature provides limited mechanistic explanations for many reported adverse reactions beyond the well-understood hepatotoxicity pathway. Finally, most safety assessments focus on chronic oral use for spasticity, with limited systematic evaluation of AEs following acute intravenous administration for MH treatment.

To address this gap, we conducted a comprehensive analysis of AEs using 3 major pharmacovigilance databases: the U.S. Food and Drug Administration Adverse Event Reporting System (FAERS), Japanese Adverse Drug Event Report (JADER), and Canada Vigilance Adverse Reaction Online Database (CVARO).^[14]^ This study aims to identify significant adverse effects of dantrolene therapy, compare adverse effects between MH and non-MH indications, highlight previously underreported AEs, and investigate potential mechanisms underlying these adverse reactions. Our findings will provide healthcare professionals with updated guidance on monitoring dantrolene-related adverse reactions, particularly in MH treatment. Additionally, our preliminary mechanistic insights establish a foundation for future research into dantrolene’s adverse effects.

## 2. Method

### 2.1. Study design and data source

In this study, FAERS, JADER, and CVARO database were used to identify AEs associated with dantrolene. FAERS data, obtained from the U.S. Food and Drug Administration website, included reports of drug-related AEs from multiple countries and regions, providing a comprehensive dataset for post-marketing drug safety analysis, JADER data was sourced from the Pharmaceuticals and Medical Devices Agency website in Japan,^[15]^ it compiled reports of drug-related AEs in Japan, while AE reports from Canada were sourced from the CVARO database. In this study, a comprehensive analysis of adverse drug reactions using 3 databases were conducted. The goal was to include as much data as possible to achieve more accurate and reliable results.

This study included all AEs related to dantrolene extracted from the 3 databases. No restrictions were placed on reporting time; therefore, the study spanned from the first recorded dantrolene-related event in the databases up to 2024. As the data used in this research were de-identified, ethical approval was not required.

### 2.2. Cases and drugs definition

In the FAERS, JADER, and CVAR databases, AEs were categorized using the System Organ Class, High Level Group Term, High Level Term, and Preferred Terms as defined by MedDRA. In this study, Preferred Terms were used for classification. To collect comprehensive data, different names for the target drug were used to query the 3 databases. Preferred suspect was designated to ensure dantrolene was the primary suspect drug, enhancing reliability by excluding events caused by other primary suspect drugs.

AEs involving dantrolene as the primary suspect were analyzed. FAERS data covered events from 2004 to 2024, JADER from 2004 to 2023, and the CVARO database from 1991 to 2019.

### 2.3. Descriptive analysis

The demographic characteristics of the patients (e.g., gender, age, weight, reporting location, and reporter category) and clinically relevant aspects (e.g., patient outcomes) were summarized in the baseline information table. It should be noted that the baseline characteristics were presented as person–times, meaning that if the same individual reported AEs in multiple years, each annual report was counted separately as an independent instance in the demographic analysis.

### 2.4. Signal screening

In this study, we utilized disproportionality analysis, a method widely used in pharmacovigilance research.^[16]^ This method includes 4 specific indices: reporting odds ratio (ROR),^[17]^ proportional reporting ratio (PRR),^[18]^ Bayesian confidence propagation neural network (BCPNN),^[19]^ and Empirical Bayesian Geometric Mean ^[20]^ These indices were calculated using standard formulas (Table S1, Supplemental Digital Content, https://links.lww.com/MD/Q840). In our study, these 4 indices were applied to the FAERS, JADER, and CVARO databases to identify positive signals for drug–AE associations. Only AEs with positive results in all 4 indices were selected as final positive signals for analyzing dantrolene-related AEs.

Additionally, important medical events (IME) were selected from the positive AEs based on the MedDRA Important Medical Event Terms List (version 27.1),^[21]^ coordinated by the EudraVigilance Expert Working Group. In the process of handling AE reports, severity was judged according to 6 criteria outlined in the Pharmacovigilance Quality Management Standards: death, life-threatening, hospitalization or extended hospitalization, permanent or significant disability or functional loss, congenital abnormalities or birth defects, and other significant medical events that could occur without treatment.

### 2.5. Time-to-onset analysis (TTO)

In this study, the time association between drug use and AEs was analyzed to identify potential high-risk windows. It is crucial to emphasize that the TTO analysis was conducted exclusively on AEs from the FAERS database, as this was the only database among the 3 pharmacovigilance databases (FAERS, JADER, CVARO) that contains complete information on both drug use and AE onset times. Therefore, the TTO analysis results represent temporal patterns specific to the FAERS database and cannot be extrapolated to other databases. This important limitation should be fully considered when interpreting the temporal patterns of dantrolene-associated AEs.

### 2.6. Subgroup analysis through age, treatment duration, and indication groups

We conducted subgroup analysis through different groups to see whether there were differences in PT between different subgroups. As there was very little data in CVARO database, those data were given up. The patients were divided through age, treatment duration, and indication. Patients are categorized by age into 18 to 45 years and ≥ 45 years, by treatment duration into ≤ 180 days and ≥ 180 days, and by indication into MH and non-MH. In this subgroup analysis, the *P* values were calculated using Fisher precise test to test for statistical differences in the incidence of AEs between the 2 groups. In the age group, ROR reflected the predominance of AEs in those aged 18 to 45 years compared with those aged over 45 years. In the treatment duration group, ROR reflected the predominance of AEs in those treated for <180 days compared with those treated for more than 180 days. In the indication group, ROR reflected the advantage of AEs in the indication group with MH compared with the indication group with non-MH.

### 2.7. Differential gene expression analysis

There were 2 study series in the Gene Expression Omnibus Datasets for differential gene expression studies which were strongly related to dantrolene use. One study focused on porcine aortic valve interstitial cells, and this study was identified as GSE227229.^[22]^ The 2 selected sample groups in this study consisted of porcine aortic valve interstitial cells treated with lysophosphatidylcholine alone versus those treated with lysophosphatidylcholine plus dantrolene to investigate the inhibitory effects of dantrolene on calcific nodule formation. The other study focused on mouse cardiac hypertrophy, and this study was identified as GSE184769. The 2 selected sample groups in this study consisted of mice that underwent transverse aortic constriction surgery and were postoperatively administered either dantrolene (30 mg/kg) or solvent control to evaluate the therapeutic effects on pressure overload-induced cardiac hypertrophy. We selected these groups based on whether dantrolene was the only variable in the intervention of the experimental subjects. The criteria for screening differentially expressed genes were set at |logFC| > 1 and *P* value < .05.

We also identified functions and pathways of differential gene enrichment using Gene Ontology (GO) Enrichment Analysis and Kyoto Encyclopedia of Genes and Genomes (KEGG) Enrichment Analysis to compare gene expression differences between groups with and without dantrolene. The screened differentially expressed genes were imported into the STRING database to construct a protein–protein interaction (PPI) network and visualized the PPI network. Also, the key genes in both PPI network were calculated using CytoHubba in Cytoscape (version 3.10.3).

### 2.8. Case by case analysis

To analyze dantrolene-associated adverse reactions in MH patients, we separately examined AEs specifically indicated for MH treatment and extracted all relevant patient data from the comprehensive database. Since the CVARO database did not include patients with the indication of MH, the case by case analysis was conducted only in the FAERS and JADER databases.

## 3. Result

### 3.1. Descriptive analysis

Figure S1, Supplemental Digital Content, https://links.lww.com/MD/Q840 illustrated the research flowchart of this study. From 1991 to 2024, 21,433,114 AEs were reported in the FAERS databases, 905,431 AEs were reported in the CVARO database, 903,522 AEs were reported in the JADER database. After deduplication, 18,182,912 reports remained in the FAERS database, 35 reports remained in the CVARO database, 240 reports remained in the JADER database. Among these, there were 274 AE reports related to dantrolene in the FAERS database, 12 AE reports related to dantrolene in the CVARO database, and 311 AE reports related to dantrolene in the JADER database. The basic information of patients was summarized in Table 1. In terms of gender distribution, in FAERS, 28.2% (N = 77) of the AE reports were for females, and 54.9% (N = 150) were for males. In CVARO, 43.8% (N = 14) of the AE reports were for females, and 53.1% (N = 17) were for males. In JADER, 32.1% (N = 77) of the AE reports were for females, and 61.3% (N = 147) were for males. Most of the weight-related data from the 3 databases were missing. The body weight distribution of patients in the 3 databases was 4.4%, 9.9%, 2.9% (N = 12/27/8, <50 kg/50 to 100 kg/>100 kg) (FAERS), 3.1%, 37.5%, 3.1% (N = 1/12/1, <50 kg/50 to 100 kg/>100 kg) (CVARO), 16.2%, 17.1% (N = 39/41, <50 kg/50 to 100 kg) (JADER). In terms of age, patients in the 3 databases were mainly aged 18 to 64 years (46.2%/81.3%/57.5%, FEARS/CVARO/JADER), followed by 64 to 85 years (13.9%/6.3%/20%, FAERS/CVARO/JADER). In the FAERS database, the majority of AE reports came from Medical Doctors (30.8%, N = 84), Consumers (26.4%, N = 72), and Pharmacists (20.1%, N = 55). In the CVARO database, most AE reports came from Pharmacists (50.0%, N = 16). In the JADER database, the majority of AE reports came from Medical Doctors (42.1%, N = 101) and Pharmacists (42.1%, N = 101). Regarding outcome-related data, in FAERS, 10.6% (N = 29) of the AE reports resulted in death. The proportion of AE reports resulting in death in CVARO and JADER were 25.6% (N = 5) and 9.4% (N = 35), respectively.

**Table 1:** Basic information of reports from FAERS, CVARO, and JADER associated with dantrolene.

	FAERS	CVARO	JADER
	N = 273	N = 32	N = 240
Age (yr)			
<18	30 (11.0%)	0 (0%)	31 (12.9%)
18–64	126 (46.2%)	26 (81.3%)	138 (57.5%)
65–85	38 (13.9%)	2 (6.3%)	48 (20.0%)
>85	3 (1.1%)	3 (9.4%)	4 (1.7%)
Missing	76 (27.8%)	1 (3.1%)	19 (7.9%)
Sex			
Female	77 (28.2%)	14 (43.8%)	77 (32.1%)
Male	150 (54.9%)	17 (53.1%)	147 (61.3%)
Missing	46 (16.8%)	1 (3.1%)	16 (6.7%)
Weight (kg)			
<50	12 (4.4%)	1 (3.1%)	39 (16.2%)
50–100	27 (9.9%)	12 (37.5%)	41 (17.1%)
>100	8 (2.9%)	1 (3.1%)	0 (0%)
Missing	226 (82.8%)	18 (56.3%)	160 (66.7%)
Reporter country			
United States	92 (33.7%)	–	–
Japan	51 (18.7%)	–	240 (100%)
Netherland	42 (15.4%)	–	–
Country not specified	12 (4.4%)	–	–
France	11 (4.0%)	–	–
United Kingdom	11 (4.0%)	–	–
Canada	7 (2.6%)	32 (100%)	–
Others	47 (17.2%)	–	–
Reporter			
Consumer	72 (26.4%)	2 (6.3%)	26 (10.8%)
Health professional	5 (1.8%)	5 (15.6%)	10 (4.2%)
Medical doctor	84 (30.8%)	2 (6.3%)	101 (42.1%)
Others	41 (15.0%)	0 (0%)	0 (0%)
Pharmacist	55 (20.1%)	16 (50.0%)	101 (42.1%)
Missing	16 (5.9%)	7 (21.9%)	2 (0.8%)
Outcome			
Death	29 (10.6%)	5 (15.6%)	35 (9.4%)
Disability	2 (0.7%)	–	–
Hospitalization	73 (26.7%)	13 (40.6%)	–
Life-threatening	27 (9.9%)	3 (9.4%)	–
Other serious illness	108 (39.6%)	–	–
Not serious	–	9 (28.1%)	–
Other medically important condition	–	2 (6.3%)	–
Sequelae	–	–	8 (2.1%)
Recovered	–	–	130 (34.9%)
Mild recovered	–	–	83 (22.3%)
Not recovered	–	–	27 (7.2%)
Missing	34 (12.5%)	–	90 (24.1%)

All data are shown as n (%).

CVARO = Canada Vigilance Adverse Reaction Online Database, FAERS = U.S. Food and Drug Administration Adverse Event Reporting System, JADER = Japanese Adverse Drug Event Report.

**Figure 1.**
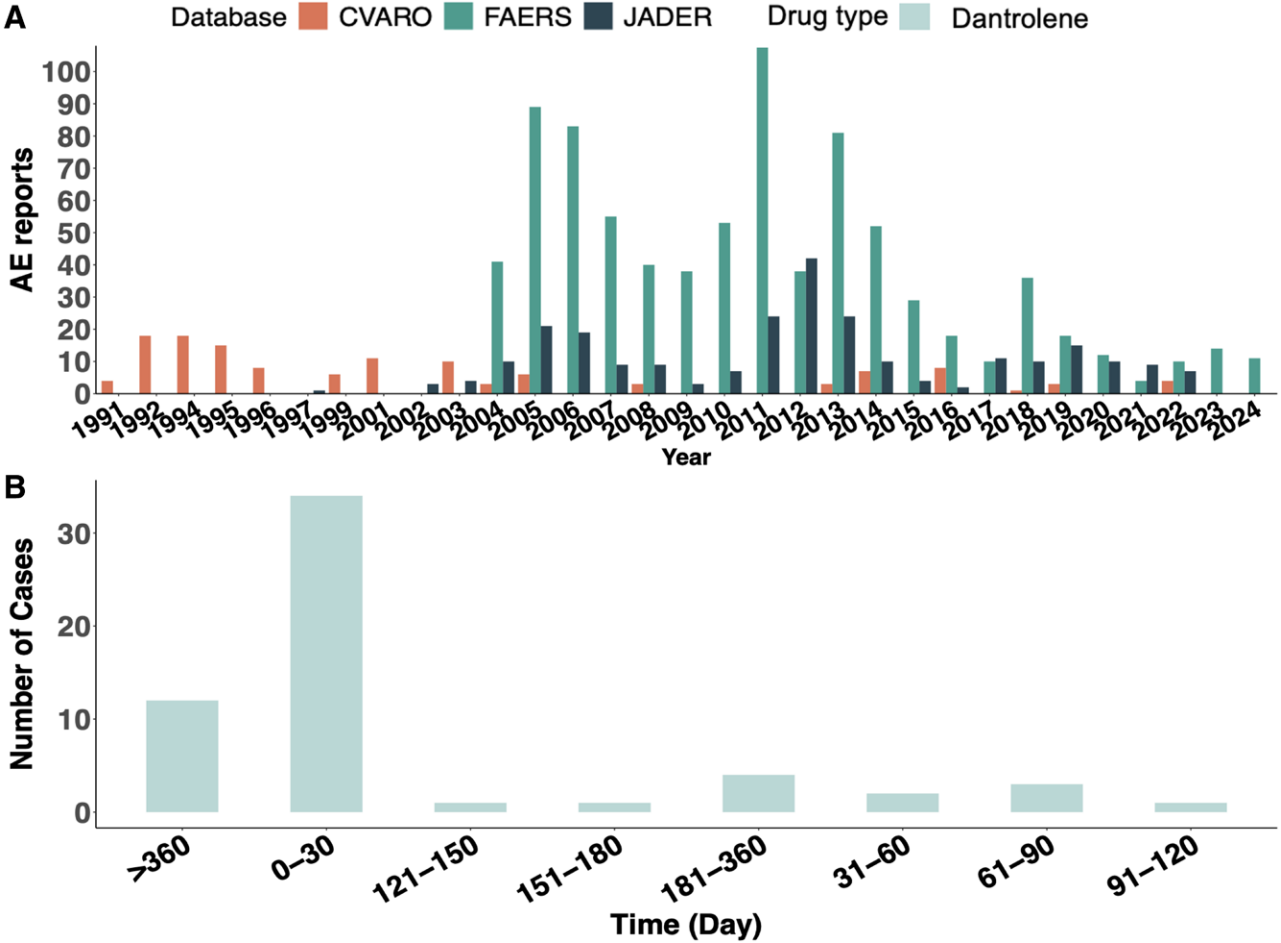
Comprehensive analysis of dantrolene. (A) Frequency of dantrolene’s AEs in FAERS, JADER, and CVARO database (1991–2024). (B) The frequency of AEs occurred in different time periods in FAERS database (2004–2024). AEs = adverse events, CVARO = Canada Vigilance Adverse Reaction Online Database, FAERS = U.S. Food and Drug Administration Adverse Event Reporting System, JADER = Japanese Adverse Drug Event Report.

**Figure 2.**
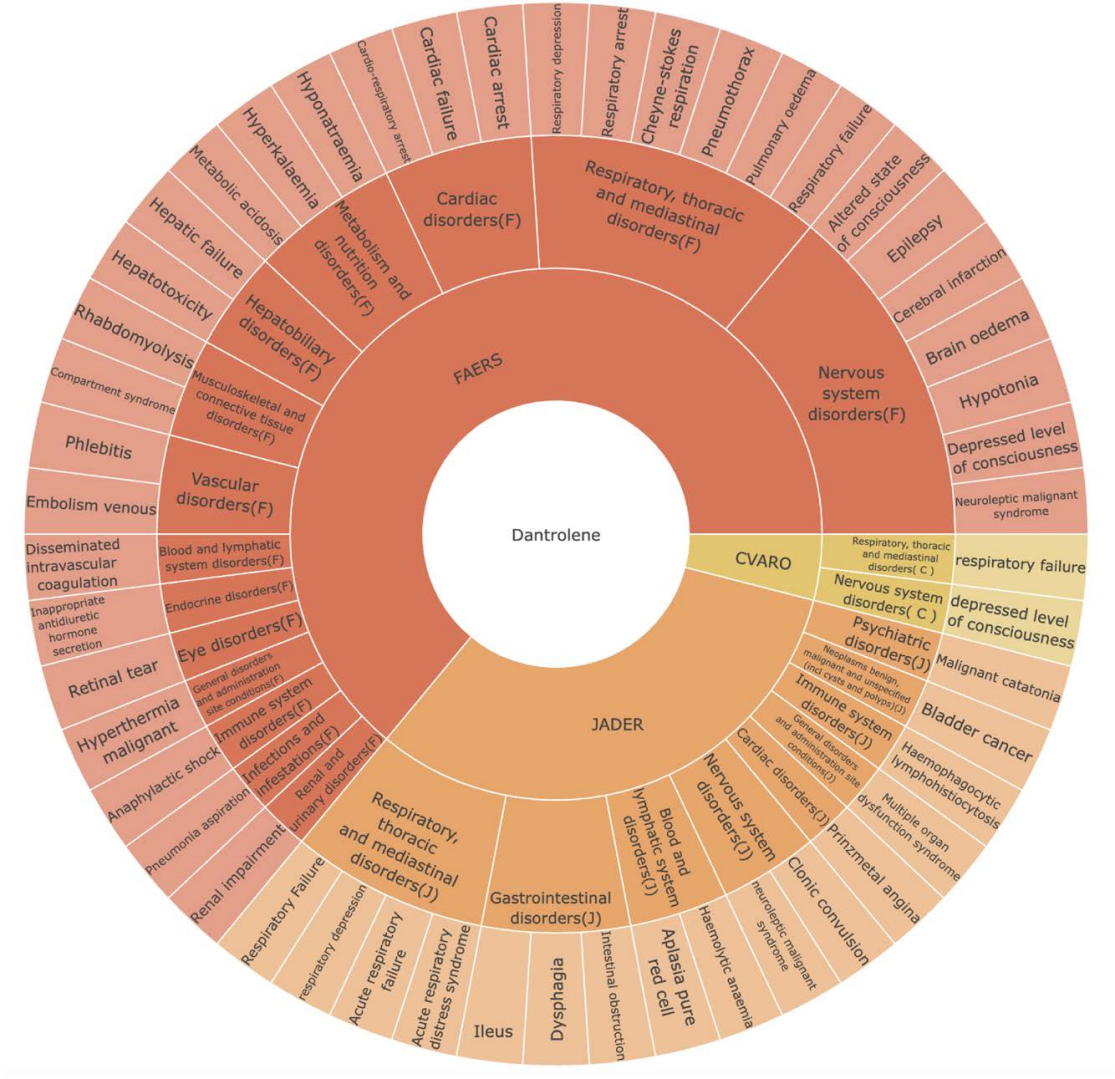
Classification of the AEs related to dantrolene in FAERS, JADER, and CVARO database. AEs = adverse events, CVARO = Canada Vigilance Adverse Reaction Online Database, FAERS = U.S. Food and Drug Administration Adverse Event Reporting System, JADER = Japanese Adverse Drug Event Report.

Figure 1A showed the variation in the number of dantrolene reports from 1991 to 2024. In FAERS, the AE reports were mainly concentrated between 2004 and 2024, which gradually decreased since 2005, gradually increased after 2010, and showed a downward trend since 2013. In CVARO, the AE reports were primarily concentrated between 1992 and 1996, the 5-year average was 15 cases, with fewer reports in the years thereafter, with 5 reports average in these years, In JADER, the AE reports were mainly concentrated between 2005 and 2022. Just like FAERS, AE reports decreased after 2005, gradually increased after 2010, with an overall downward trend since 2012. This reflects the evolution of pharmacovigilance systems from volume-driven to quality-focused reporting, influenced by regulatory policy changes and improved signal detection methods. Additionally, enhanced MH management protocols after 2010, including standardized dantrolene dosing guidelines, contributed to more accurate AE reporting. Since only the FAERS database reported the onset time of related AE reports, Figure 1B summarized the onset times of AE reports from FAERS. The data showed that the onset times were primarily concentrated within 0 to 30 days and over 180 days. The use of dantrolene was mainly associated with timely adverse reactions and long-term effects, so close postoperative monitoring and long-term follow-up are required. All the concrete data that appeared in Figure 2 was displayed in Table S2, Supplemental Digital Content, https://links.lww.com/MD/Q840.

### 3.2. Dantrolene-related AEs

In the selected dantrolene-related positive AE reports, we filtered the IMEs based on the MedDRA Important Medical Event Terms List (version 27.1). There were 14 categories with 32 AEs from FAERS, 2 categories with 2 AEs from CVARO, and 9 categories with 16 AEs from JADER. Figure 2 categorized and displayed this information. Respiratory, thoracic, and mediastinal disorders and nervous system disorders can all be found in 3 databases. What’s more, blood and lymphatic system disorders, cardiac disorders, and immune system disorders were exclusively found in FAERS and JADER databases.

**Figure 3.**
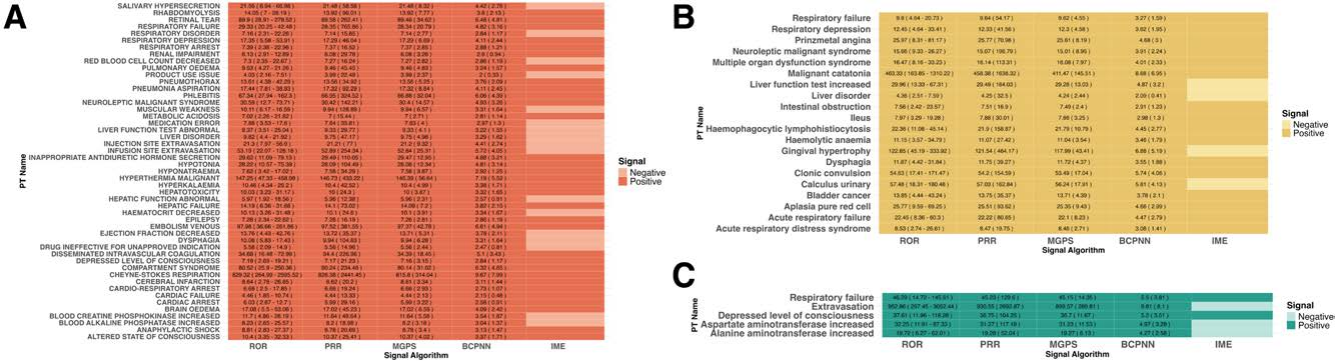
The heatmap of dantrolene in FAERS, JADER, and CVARO database and. (A) The heatmap of dantrolene in FAERS database. (B) The heatmap of dantrolene in JADER database. (C) The heatmap of dantrolene in CVARO database. CVARO = Canada Vigilance Adverse Reaction Online Database, FAERS = U.S. Food and Drug Administration Adverse Event Reporting System, JADER = Japanese Adverse Drug Event Report.

We used all the selected positive AE reports from the 3 databases to create a heatmap (Fig. 3A and B). Hepatobiliary disorder, a widely recognized adverse effect from the use of dantrolene, appeared in all 3 databases. The FAERS database contained liver function test abnormal, liver disorder, hepatotoxicity, hepatic function abnormal, and hepatic failure, the JADER database contained Liver function test increased and Liver disorder, the CVARO database contained aspartate aminotransferase increased and alanine aminotransferase increased. However, only hepatic failure (ROR, 14.19 [6.36–31.68]; PRR, 14.1 [73.02]; MGPS, 14.09 [7.2]; BCPNN, 3.82 [2.15]) and hepatotoxicity (ROR, 10.03 [3.23–31.17]; PRR, 10 [24.3]; MGPS, 10 [3.87]; BCPNN, 3.32 [1.65]) mentioned in the FAERS database were IMEs. Respiratory, thoracic, and mediastinal disorders appeared in all 3 databases and were the major ones. Respiratory failure was common to all 3 databases and was an IME, with ROR 29.33 (20.25–42.48)/9.8 (4.64–20.73)/46.29 (14.72–145.61) (FAERS/JADER/CVARO), PRR 28.35 (765.86)/9.64 (54.17)/45.23 (129.6) (FAERS/JADER/CVARO), MGPS 28.34 (20.79)/9.62 (4.55)/45.15 (14.35) (FAERS/JADER/CVARO), BCPNN 4.82 (3.16)/3.27 (1.59)/5.5 (3.81) (FAERS/JADER/CVARO). The category musculoskeletal and connective tissue appears only in the FAERS database and includes rhabdomyolysis, compartment syndrome, and muscular weakness. Rhabdomyolysis (ROR, 14.05 (7–28.19); PRR, 13.92 (96.01); MGPS, 13.92 (7.77); BCPNN, 3.8 (2.13)) and compartment syndrome (ROR, 80.52 (25.9–250.36); PRR, 80.24 (234.48); MGPS, 80.14 (31.02); BCPNN, 6.32 (4.65)) was the IMEs.

**Figure 4.**
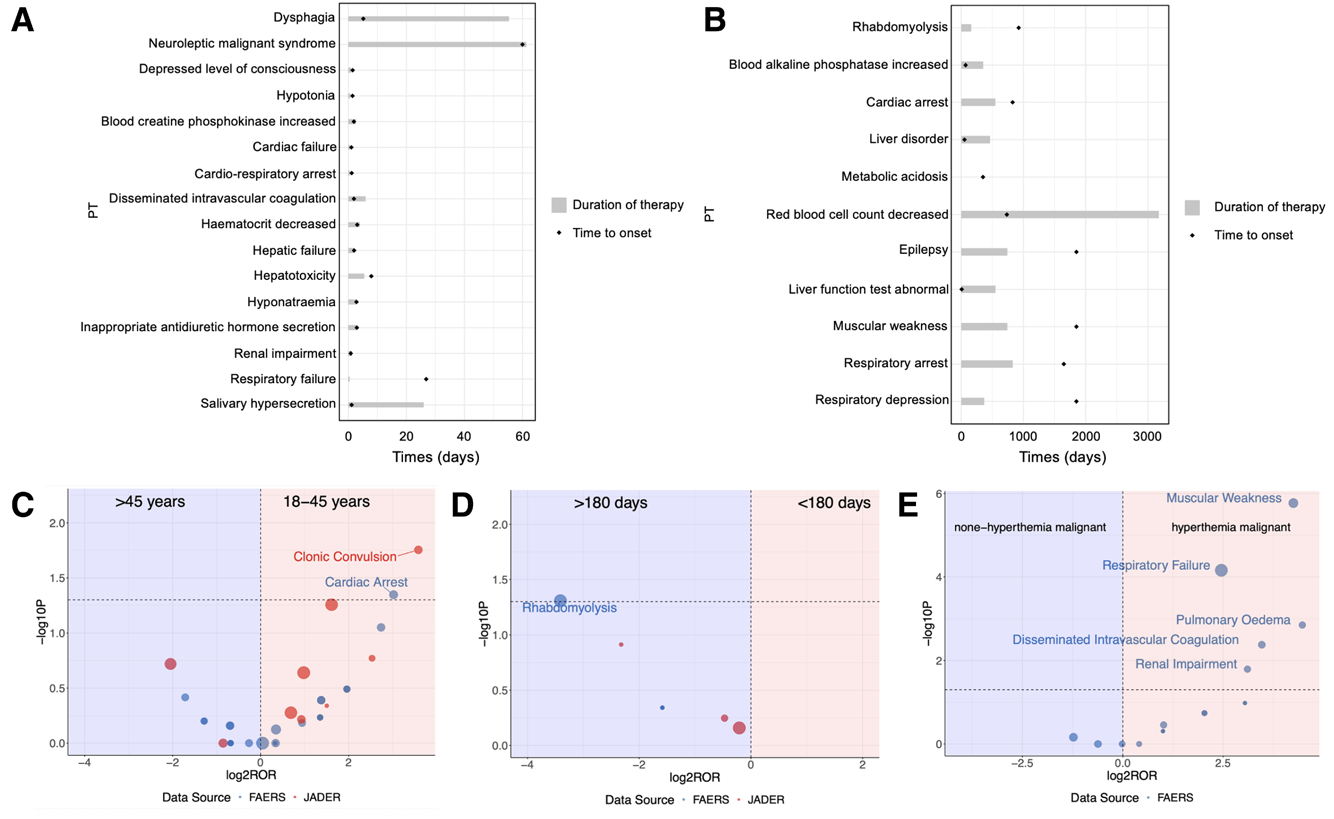
The time-to-onset analysis of dantrolene in FAERS database and subgroup analysis of dantrolene in FAERS and JADER database. (A) The time-to-onset analysis of dantrolene in FAERS database (onset times shorter than 100 days). (B) The time-to-onset analysis of dantrolene in FAERS database (onset times longer than 100 days). (C) Subgroup analysis of dantrolene through ages in FAERS and JADER database. (D) Subgroup analysis of dantrolene through treatment durations in FAERS and JADER database. (E) Subgroup analysis of dantrolene through indications in FAERS database. FAERS = U.S. Food and Drug Administration Adverse Event Reporting System, JADER = Japanese Adverse Drug Event Report.

Due to significant variations in the treatment and onset times across different cases, we categorized the cases into those with treatment and onset times shorter than 100 days and those longer than 100 days, displaying them in 2 separate graphs (Fig. 4A and B). The AE whose onset time shorter than 100 days such as dysphagia, NMS, depressed level of consciousness, hypotonia, blood creatine phosphokinase increased, cardiac failure, cardio-respiratory arrest, disseminated intravascular coagulation, hematocrit decreased, hepatic failure, hepatotoxicity, hyponatremia, inappropriate antidiuretic hormone secretion, renal impairment, respiratory failure, salivary hypersecretion. AEs with onset times exceeding 100 days included rhabdomyolysis, increased blood alkaline phosphatase, cardiac arrest, liver disorder, metabolic acidosis, decreased red blood cell count, epilepsy, abnormal liver function tests, muscular weakness, respiratory arrest, and respiratory depression. In some cases, AEs occurred during the treatment period, while in others, they occurred after treatment. Among the cases with AEs occurring after treatment, some did not show any adverse reactions until more than 2 years after treatment.

### 3.3. Subgroup analysis through age, treatment duration, and indication

The volcano plot of age groups (Fig. 4C) revealed that cardiac arrest (ROR (95% CI) 8.12 (0.95–69.00); *P* value .045) was discrepant among 2 age groups in FAERS database and clonic convulsion (ROR (95% CI) 3.58 (1.31–109.75); *P* value .0178) in JADER database. Both adverse reactions were more likely to occur in people aged 18 to 45 years. In the volcano plot of treatment duration groups (Fig. 4D), rhabdomyolysis in (ROR (95% CI) 0.09 (0.01–1.00); *P* value .049) FAERS database was the only one which showed the difference between 2 treatment groups. This adverse reaction was more likely to occur when the treatment duration was <180 days. In the JADER database, subgroup analysis related to indication grouping did not yield significant results. Therefore, the volcano plot for indications (Fig. 4E) only displayed the analysis results from the FAERS database. Muscular weakness (ROR (95% CI) 19.00 (5.03–71.83); *P* value < .001), respiratory failure (ROR (95% CI) 5.48 (2.42–12.42); *P* value < .001), pulmonary edema (ROR (95% CI) 22.18 (2.52–195.23); *P* value .001), disseminated intravascular coagulation (ROR (95% CI) 11.03 (2.06–58.94); *P* value .004) and renal impairment (ROR (95% CI) 8.60 (1.52–48.60); *P* value .016) were discrepant between non-hyperthermia malignant and hyperthermia malignant. In this subgroup analysis, all the above adverse effects were more likely to occur during the treatment of MH.

### 3.4. Differential expression of dantrolene-related genes

**Figure 5.**
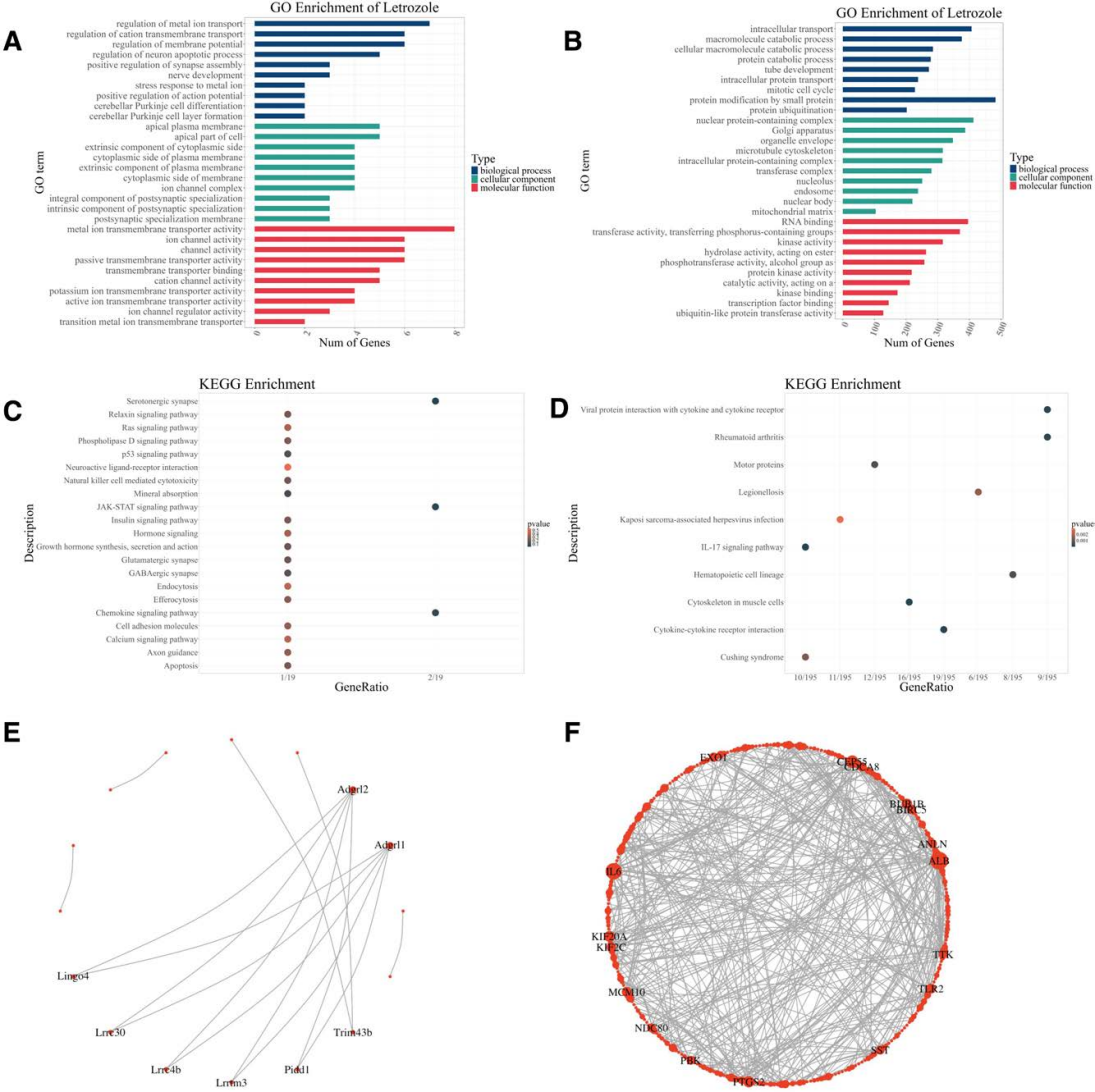
Differential expression of dantrolene-related genes. (A) GO analysis of GSE184769. (B) GO analysis of GSE227229. (C) KEGG analysis of GSE184769. (D) KEGG analysis of GSE227229. (E) PPI network of GSE184769. (F) PPI network of GSE227229. GO = Gene Ontology, KEGG = Kyoto Encyclopedia of Genes and Genomes, PPI = protein–protein interaction.

In the GEO datasets GSE184769 and GSE227229, we selected 116 and 561 DEGs. GO enrichment analysis of these DEGs were shown in Figure 5A and B. KEGG enrichment analysis of these DEGs were shown in Figure 5C and D. Through systematic interpretation of the GO and KEGG enrichment analysis results, we found that drug treatment significantly affected key pathways in multiple physiological systems. In terms of skeletal muscle function, the enrichment of ion channel complexes, potassium transmembrane transporter activity, and calcium signaling pathways indicated their direct regulatory role in muscle contraction function. Meanwhile, the activation of stress response to metal ions and macromolecule catabolic processes may be associated with fever and malaise symptoms. Dantrolene-induced liver injury is also reflected herein. Dantrolene primarily caused liver-related adverse reactions through oxidative stress and metal ion metabolism disorder, apoptosis, and toxic accumulation, metabolic burden and abnormal protein catabolism, such as stress response to metal ion, apoptosis, protein catabolic process.

The PPI network (Fig. 5E and F) showed the interaction between these DEGs, the key genes calculated by cytohubba in GSE184769 were Adgrl1 and Adgrl2, while the key genes in GSE227229 were IL6 and ALB.

### 3.5. Case by case analysis

The relevant data of patients with MH as the indication were extracted from the FAERS (Table S3, Supplemental Digital Content, https://links.lww.com/MD/Q840) and JADER (Table S4, Supplemental Digital Content, https://links.lww.com/MD/Q840) databases. During the use of dantrolene in patients with MH, in addition to the adverse reactions identified in subgroup analyses, other AEs were observed in some patients. Notably, ocular-related adverse reactions were reported, including retinal detachment, retinal tear, elevated intraocular pressure, and monocular or binocular blindness. Furthermore, coagulation-related AEs such as deep vein thrombosis, disseminated intravascular coagulation, and brachiocephalic vein thrombosis also warrant special attention.

## 4. Discussion

Through comprehensive analysis of pharmacovigilance databases and molecular studies, our research revealed several significant AEs associated with dantrolene use. Most notably, we identified AEs in both respiratory and musculoskeletal systems, including respiratory failure in younger patients and severe musculoskeletal complications such as compartment syndrome and rhabdomyolysis. These AEs were particularly pronounced in patients with MH. Molecular analysis identified IL6 and ALB as key mediators of respiratory effects, while Adgrl1 and Adgrl2 emerged as crucial regulators of muscle function, potentially underlying these observed AEs.

Respiratory AEs emerged as major concerns, with respiratory failure being consistently reported across all databases (ROR: 29.33–46.29). These findings align with previous reports of dantrolene-induced respiratory complications.^[23]^ During oral administration, dantrolene may cause respiratory manifestations such as dyspnea and tachypnea.^[24]^ In contrast, intravenous injection can lead to more severe respiratory adverse effects, including respiratory failure in some patients.^[25]^ From an anesthetic perspective, these findings necessitate careful cardiorespiratory monitoring and potential modification of extubation criteria. Molecular studies have demonstrated that IL6 upregulation activates inflammatory cascades in respiratory tissues through the JAK–STAT pathway, while alterations in albumin levels affect membrane potential and fluid homeostasis.^[26]^ In respiratory tissue, elevated IL6 levels not only induce inflammatory responses but also affect pulmonary vascular permeability.^[27]^ The dysregulation of albumin levels directly correlates with pulmonary edema formation through altered fluid balance mechanisms,^[28]^ while the inflammatory cascade significantly impairs alveolar fluid clearance mechanisms, exacerbating tissue damage when severe edema is present (wet/dry ratio > 6.5).^[29]^

Musculoskeletal demonstrated distinct patterns, with rhabdomyolysis (ROR: 14.05) and compartment syndrome (ROR: 80.52) being particularly concerning. The use of dantrolene is commonly associated with muscle weakness due to calcium ion homeostasis disturbance, which may lead to dysphagia in postoperative patients. Additionally, dyspnea, a respiratory adverse effect, is also linked to dantrolene-induced muscle weakness.^[30]^ Our molecular analysis revealed t mechanisms through ADGRL1 and ADGRL2 in muscular tissues. In muscle tissue, ADGRL1 demonstrates specific localization at neuromuscular junctions, where it plays a crucial role in synaptogenesis and signal transmission through multiple signaling pathways.^[31]^ ADGRL1 haploinsufficiency can lead to severe neurodevelopmental disorders and altered synaptic activity, particularly affecting neuromuscular junction formation and function.^[32]^ While both proteins share sequence similarity, they exhibit distinct tissue expression patterns – ADGRL1 shows prominent expression in cardiac tissue during development, whereas ADGRL2 demonstrates specific expression patterns in neurons and at neuromuscular junctions.

MH patients exhibited markedly increased severity of AEs compared to non-MH indications. MH patients showed significantly higher risks of muscular weakness (ROR: 19.00), respiratory failure (ROR: 5.48), and pulmonary edema (ROR: 22.18). During anesthesia, early recognition of MH signs is crucial, as research has demonstrated that every 30-minute delay in dantrolene administration significantly increases the risk of complications, with complication rates increasing 1.6-fold per 30-minute delay.^[33]^ Dantrolene targets the mutant RYR1 gene, which plays a key role in MH pathogenesis. The RYR gene family includes 3 members: RYR1, predominantly expressed in skeletal muscle sarcoplasmic reticulum; RYR2, mainly found in cardiomyocyte sarcoplasmic reticulum; and RYR3, primarily present in brain and smooth muscle tissues. Research on RYR3 mutations remains limited, though it enhances RYR1-mediated calcium release in neonatal skeletal muscle.^[34]^ Molecular investigations of RYR1 variants demonstrate that different mutations affect calcium homeostasis and drug sensitivity through modifications of existing regulatory mechanisms.^[35]^ This understanding is further supported by detailed structural studies showing that dantrolene’s binding site remains consistent, while the protein’s conformational changes due to mutations may alter the efficiency of this interaction.^[36]^ These mutated channels show increased sensitivity to dantrolene’s calcium-modulating effects, leading to excessive suppression of calcium signaling. This heightened sensitivity amplifies IL6-mediated inflammation and Adgrl1/2 dysfunction, particularly affecting neuromuscular junction stability and respiratory muscle function.^[37,38]^ This molecular mechanism explains the observed higher incidence of AEs in MH patients and suggests the need for personalized dosing strategies.

Several limitations warrant consideration. First, temporal coverage varied among databases, potentially affecting AE detection rates. Second, substantial missing weight-related data limited subgroup analyses. Third, time-to-onset analysis was restricted to FAERS data, potentially missing important temporal patterns in other populations. Fourth, gene expression analysis was based on only 2 datasets, which may not capture the full spectrum of molecular mechanisms. Fifth, missing data on administration routes prevented analysis of route-specific AEs, which may influence both the type and severity of reactions. Sixth, potential confounders including patient comorbidities, concomitant medications, and underlying disease severity were not fully captured in the databases, potentially affecting the observed associations. Seventh, reporting bias inherent in spontaneous reporting systems may lead to underreporting of mild AEs and overreporting of severe or unexpected events, particularly in MH patients who receive closer monitoring. Eighth, the retrospective nature of database analyses limits our ability to establish definitive causal relationships between dantrolene exposure and observed AEs.

## 5. Conclusion

In conclusion, while respiratory and musculoskeletal adverse effects of dantrolene are well-established, our study provides novel insights into differential patient susceptibility. Most significantly, we demonstrated that MH patients exhibit markedly increased AE severity compared to non-MH patients, with elevated risks of muscular weakness (ROR: 19.00), respiratory failure (ROR: 5.48), and pulmonary edema (ROR: 22.18). Our molecular analysis revealed that mutant RYR1 channels in MH patients show excessive sensitivity to dantrolene’s calcium-modulating effects, amplifying IL6-mediated inflammation and disrupting Adgrl1/2-regulated neuromuscular function. This heightened molecular sensitivity explains the increased AE incidence in MH patients and represents the key novel finding of our research. These results emphasize the critical need for MH-specific dosing strategies and enhanced monitoring protocols in clinical practice.

## Acknowledgments

We gratefully acknowledge the DeepSeek for its intelligent language polishing service, which significantly improved the clarity and fluency of our manuscript. We also extend our sincere thanks to Professor Juan Ni for her invaluable guidance on the study design and throughout the manuscript revision process. Furthermore, the drug-related adverse event data utilized in this research were sourced from 3 publicly available databases: FAERS, JADER, and CVARO. We sincerely thank these platforms for their open-access data sharing.

## Author contributions

**Conceptualization**: Haohui Chen, Xulin Zhang.

**Formal analysis**: Dongxu Chen, Xiaoqin Jiang, Chao Yu.

**Funding acquisition**: Dongxu Chen, Xiaoqin Jiang, Chao Yu.

**Methodology**: Haohui Chen, Xulin Zhang, Dongxu Chen, Xiaoqin Jiang, Chao Yu.

**Project administration**: Haohui Chen, Xulin Zhang.

**Software**: Dongxu Chen, Xiaoqin Jiang, Chao Yu.

**Supervision**: Haohui Chen, Xulin Zhang.

**Writing – original draft**: Dongxu Chen, Xiaoqin Jiang, Chao Yu.

**Writing – review & editing**: Haohui Chen, Xulin Zhang, Dongxu Chen, Xiaoqin Jiang, Chao Yu.

## Supplementary Material

## References

[1] Ketel WB Kolb ME. Long-term treatment with dantrolene sodium of stroke patients with spasticity limiting the return of function. Curr Med Res Opin. 1984;9:161–9. 6499510 10.1185/03007998409109576

[2] Anderson DN. Treating depression in old age: the reasons to be positive. Age Ageing. 2001;30:13–7. 10.1093/ageing/30.1.13 11322666

[3] Harrison GG. Control of the malignant hyperpyrexic syndrome in MHS swine by dantrolene sodium. Br J Anaesth. 1975;47:62–5. 1148076 10.1093/bja/47.1.62

[4] Rosenberg MR Green M. Neuroleptic malignant syndrome. Review of response to therapy. Arch Intern Med. 1989;149:1927–31. 2673115 10.1001/archinte.149.9.1927

[5] Marelli A Bodini P Dizioli P Chiodelli G Guarneri M Boldori L. The neuroleptic malignant syndrome (NMS). A report of a clinical case with a protracted and recurrent course. A review of the literature. Minerva Med. 1996;87:45–51. 8610025

[6] Snyder HR Jr Davis CS Bickerton RK Halliday RP. 1-[(5-arylfurfurylidene)amino]hydantoins. A new class of muscle relaxants. J Med Chem. 1967;10:807–10. 6048486 10.1021/jm00317a011

[7] Lietman PS Haslam RH Walcher JR. Pharmacology of dantrolene sodium in children. Arch Phys Med Rehabil. 1974;55:388–92. 4853611

[8] Rosenberg H Pollock N Schiemann A Bulger T Stowell K. Malignant hyperthermia: a review. Orphanet J Rare Dis. 2015;10:93. 26238698 10.1186/s13023-015-0310-1 PMC4524368

[9] Halliday NJ. Malignant hyperthermia. J Craniofac Surg. 2003;14:800–2. 14501352 10.1097/00001665-200309000-00039

[10] Larach MG Brandom BW Allen GC Gronert GA Lehman EB. Cardiac arrests and deaths associated with malignant hyperthermia in North America from 1987 to 2006: a report from the North American malignant hyperthermia registry of the malignant hyperthermia association of the United States. Anesthesiology. 2008;108:603–11. 18362591 10.1097/ALN.0b013e318167aee2

[11] Larach MG Klumpner TT Brandom BW. Succinylcholine use and dantrolene availability for malignant hyperthermia treatment: database analyses and systematic review. Anesthesiology. 2019;130:41–54. 30550426 10.1097/ALN.0000000000002490

[12] Grunau BE Wiens MO Brubacher JR. Dantrolene in the treatment of MDMA-related hyperpyrexia: a systematic review. CJEM. 2010;12:435–42. 20880437 10.1017/s1481803500012598

[13] Utili R Boitnott JK Zimmerman HJ. Dantrolene-associated hepatic injury. Incidence and character. Gastroenterology. 1977;72:610–6. 838213

[14] Tse L Barr AM Scarapicchia V Vila-Rodriguez F. Neuroleptic malignant syndrome: a review from a clinically oriented perspective. Curr Neuropharmacol. 2015;13:395–406. 26411967 10.2174/1570159X13999150424113345 PMC4812801

[15] Imai T Hazama K Kosuge Y Suzuki S Ootsuka S. Preventive effect of rebamipide on NSAID-induced lower gastrointestinal tract injury using FAERS and JADER. Sci Rep. 2022;12:2631. 35173236 10.1038/s41598-022-06611-y PMC8850592

[16] Shu Y He X Liu Y Wu P Zhang Q. A real-world disproportionality analysis of olaparib: data mining of the public version of FDA adverse event reporting system. Clin Epidemiol. 2022;14:789–802. 35789689 10.2147/CLEP.S365513 PMC9250344

[17] Rothman KJ Lanes S Sacks ST. The reporting odds ratio and its advantages over the proportional reporting ratio. Pharmacoepidemiol Drug Saf. 2004;13:519–23. 15317031 10.1002/pds.1001

[18] Evans SJ Waller PC Davis S. Use of proportional reporting ratios (PRRs) for signal generation from spontaneous adverse drug reaction reports. Pharmacoepidemiol Drug Saf. 2001;10:483–6. 11828828 10.1002/pds.677

[19] Noguchi Y Nagasawa H Tachi T Tsuchiya T Teramachi H. Signal detection of oral drug-induced dementia in chronic kidney disease patients using association rule mining and Bayesian confidence propagation neural network. Pharmazie. 2019;74:570–4. 31484600 10.1691/ph.2019.9426

[20] He L Li Q Yang Y. Pharmacovigilance study of GLP-1 receptor agonists for metabolic and nutritional adverse events. Front Pharmacol. 2024;15:1416985. 39040467 10.3389/fphar.2024.1416985 PMC11260617

[21] European Medicines Agency (EMA). EudraVigilance system overview. 2016. https://www.ema.europa.eu/en/human-regulatory-overview/research-development/pharmacovigilance-research-development/eudravigilance/eudravigilance-system-overview. Accessed November 15, 2024.

[22] Sylvester CB Amirkhosravi F Bortoletto AS West WJ 3rd Connell JP Grande-Allen KJ. Dantrolene inhibits lysophosphatidylcholine-induced valve interstitial cell calcific nodule formation via blockade of the ryanodine receptor. Front Cardiovasc Med. 2023;10:1112965. 37063962 10.3389/fcvm.2023.1112965 PMC10100588

[23] Ellis KO Wessels FL Carpenter JF. Effects of intravenous dantrolene sodium on respiratory and cardiovascular functions. J Pharm Sci. 1976;65:1359–64. 966155 10.1002/jps.2600650925

[24] Javed M Bogdanov A. Oral dantrolene and severe respiratory failure in a patient with chronic spinal cord injury. Anaesthesia. 2010;65:855–6. 20560919 10.1111/j.1365-2044.2010.06409.x

[25] Brandom BW Larach MG. TNAMH: reassessment of the safety and efficacy of dantrolene: [2002][A-1199]. Anesthesiology. 2002;96:A1199.

[26] Hamacher J Hadizamani Y Borgmann M. Cytokine-ion channel interactions in pulmonary inflammation. Front Immunol. 2017;8:1644. 29354115 10.3389/fimmu.2017.01644 PMC5758508

[27] Su X Bai C Hong Q. Effect of continuous hemofiltration on hemodynamics, lung inflammation and pulmonary edema in a canine model of acute lung injury. Intensive Care Med. 2003;29:2034–42. 14557856 10.1007/s00134-003-2017-3

[28] Wang H Wang T Yuan Z. Role of receptor for advanced glycation end products in regulating lung fluid balance in lipopolysaccharide-induced acute lung injury and infection-related acute respiratory distress syndrome. Shock. 2018;50:472–82. 29040215 10.1097/SHK.0000000000001032

[29] Beretta E Romanò F Sancini G Grotberg JB Nieman GF Miserocchi G. Pulmonary interstitial matrix and lung fluid balance from normal to the acutely injured lung. Front Physiol. 2021;12:781874. 34987415 10.3389/fphys.2021.781874 PMC8720972

[30] Locatelli F Pozzi M Avantaggiato P. Pharyngeal spasticity due to dantrolene. J Clin Pharm Ther. 2014;39:449–51. 24725261 10.1111/jcpt.12161

[31] Vezain M Lecuyer M Rubio M. A de novo variant in ADGRL2 suggests a novel mechanism underlying the previously undescribed association of extreme microcephaly with severely reduced sulcation and rhombencephalosynapsis. Acta Neuropathol Commun. 2018;6:109. 30340542 10.1186/s40478-018-0610-5 PMC6195752

[32] Knapp B Wolfrum U. Adhesion GPCR-related protein networks. Handb Exp Pharmacol. 2016;234:147–78. 27832488 10.1007/978-3-319-41523-9_8

[33] Tanwar P Naagar M Malik G. A review on malignant hyperthermia: epidemiology, etiology, risk factors, diagnosis, clinical management and treatment modalities. World J Biol Pharm Health Sci. 2022;13:138–61.

[34] Yang D Pan Z Takeshima H. RyR3 amplifies RyR1-mediated Ca2+-induced Ca2+ release in neonatal mammalian skeletal muscle. J Biol Chem. 2001;276:40210–4. 11500519 10.1074/jbc.M106944200

[35] Gaburjakova J Gaburjakova M. Molecular aspects implicated in dantrolene selectivity with respect to ryanodine receptor isoforms. Int J Mol Sci. 2023;24:5409. 36982484 10.3390/ijms24065409 PMC10049336

[36] Nelson TE. Malignant hyperthermia: a pharmacogenetic disease of Ca++ regulating proteins. Curr Mol Med. 2002;2:347–69. 12108947 10.2174/1566524023362429

[37] Kandel S Adhikary P Li G Cheng K. The TIM3/Gal9 signaling pathway: an emerging target for cancer immunotherapy. Cancer Lett. 2021;510:67–78. 33895262 10.1016/j.canlet.2021.04.011 PMC8168453

[38] Donohue JD Amidon RF Murphy TR. Parahippocampal latrophilin-2 (ADGRL2) expression controls topographical presubiculum to entorhinal cortex circuit connectivity. Cell Rep. 2021;37:110031. 34818557 10.1016/j.celrep.2021.110031

