# Supplementary material for: Systematic analysis of adverse reactions associated with dantrolene treatment: From clinical features to molecular mechanisms
Source: Medicine (Baltimore). 2026 May 12;104(49):e46304. doi: 10.1097/MD.0000000000046304 (PMC12688843; doi:10.1097/MD.0000000000046304)
Supplement: Supplementary file 1 [file medi-104-e46304-s001.pdf]

sTable1. Calculational formulas of ROR, PRR, BCPNN and MGPS

Calculational formulas of ROR:

$$ROR = \frac{a/c}{b/d} = \frac{ad}{bc}$$

$$SE(\ln ROR) = \sqrt{\left(\frac{1}{a}+\frac{1}{b}+\frac{1}{c}+\frac{1}{d}\right)}$$

$$95\%CI = \mathrm{e}\ln(ROR) \pm 1.96\sqrt{\left(\frac{1}{a}+\frac{1}{b}+\frac{1}{c}+\frac{1}{d}\right)}$$

Calculational formulas of PRR:

$$PRR = \frac{a/(a+b)}{c/(c+d)}$$

$$SE(\ln PRR) = \sqrt{\left(\frac{1}{a}-\frac{1}{a+b}+\frac{1}{c}-\frac{1}{c+d}\right)}$$

$$95\%CI = \mathrm{e}\ln(PRR) \pm 1.96\sqrt{\left(\frac{1}{a}-\frac{1}{a+b}+\frac{1}{c}-\frac{1}{c+d}\right)}$$

Calculational formulas of BCPNN:

$$IC = \log_2\frac{\mathrm{p}(x,y)}{\mathrm{p}(x)\mathrm{p}(y)} = \log_2\frac{a(a+b+c+d)}{(a+b)(c+d)}$$

$$E(IC) = \log_2\frac{(a+\gamma 11)(a+b+c+d+\alpha)(a+b+c+d+\beta)}{(a+b+c+d+\gamma)(a+b+\alpha 1)(a+c+\beta 1)}$$

$$V(IC) = \frac{1}{(\ln 2)^2}\left\{\left[\frac{(a+b+c+d)-\alpha+\gamma-\gamma 11}{(a+\gamma 11)(1+a+b+c+d+\gamma)}\right]+\left[\frac{(a+b+c+d)-(a+b)+\alpha-\alpha 1}{(a+b+\alpha 1)(1+a+b+c+d+\alpha)}\right]\right.$$
$$\left.+\left[\frac{(a+b+c+d)-(a+c)+\beta-\beta 1}{(a+c+\beta 1)(1+a+b+c+d+\beta)}\right]\right\}$$

$$\gamma = \gamma 11\frac{(a+b+c+d+\alpha)(a+b+c+d+\beta)}{(a+b+\alpha 1)(a+c+\beta 1)}$$

$$IC-2SD = E(IC) - 2\sqrt{V(IC)}$$

$$\alpha 1 = \beta 1 = 1\ ;\ \alpha = \beta = 2\ ;\ \gamma 11 = 1$$

Calculational formulas of MGPS:

$$EBGM = a(a+b+c+d)/(a+c)(a+b)$$

$$EBGM05 = e^{\ln(EBGM)-1.64(1/a+1/b+1/c+1/d)\text{^}0.5}$$

item	target adverse reaction reports	other adverse reaction reports	total
target drug	a	b	a+b
other drugs	c	d	c+d
total	a+c	b+d	a+b+c+d

sTable2. Data in Figure 2. Comprehensive analysis of target drugs' data.

(A) Frequency of target drugs' AEs in the FAERS, CVARO and JADER database

GetDataYear	FAERS	CVARO	JADER
1991	0	4	0
1992	0	18	0
1994	0	18	0
1995	0	15	0
1996	0	8	0
1997	0	0	1
1999	0	6	0
2001	0	11	0
2002	0	0	3
2003	0	10	4
2004	41	3	10
2005	89	6	21
2006	83	0	19
2007	55	0	9
2008	40	3	9
2009	38	0	3
2010	53	0	7
2011	108	0	24
2012	38	0	42
2013	81	3	24
2014	52	7	10
2015	29	0	4
2016	18	8	2
2017	10	0	11
2018	36	1	10
2019	18	3	15
2020	12	0	10
2021	4	0	9
2022	10	4	7

2023	14	0	0
2024	11	0	0

(B) The frequency of AEs occurred in different time periods in FAERS database.

group (days)	Dantrolene
0-30	34
31-60	2
61-90	3
91-120	1
121-150	1
151-180	1
181-360	4
>360	12

sTable3. case by case of FAERS database in malignant hyperthermia patients.

primaryid	CASEID	FDA_DT	EVENT_DT	AGE	AGE_COD	WT	WT_COD	SEX	OCCP_COD	REPORTER_COUNTRY	GetDataYear	OUTC_COD	DRUGNAME	PT
103682401	10368240	20140805	NA	36	YR	115	KG	M	MD	US	143	OT	DANTRIUM, SCOPOLAMINE, MIDAZOLAM, FENTANYL, PROPOFOL, VECURONIUM, DEXAMETHASONE, HYDROMORPHONE, KETAMINE, SODIUM BICARBONATE, CALCIUM CHLORIDE, CRYSTALLOID BOLUS	BODY TEMPERATURE INCREASED, DEEP VEIN THROMBOSIS, HEART RATE INCREASED
118162051	11816205	20151209	NA	49	YR	195	KG	M	PH	US	154	DE	RYANODEX, PROSTACYCLIN	DISEASE PROGRESSION, HYPERTHERMIA MALIGNANT
121790701	12179070	20160315	20151008	NA	NA	NA	NA	NA	CN	COUNTRY NOT SPECIFIED	161	NA	DANTRIUM, REGLAN	INFUSION SITE DISCOLOURATION, INFUSION SITE EXTRAVASATION
138601701	13860170	20170811	20170523	6	YR	NA	NA	M	PH	US	173	NA	RYANODEX	NA
141321501	14132150	20171027	NA	36	YR	115	KG	M	NA	COUNTRY NOT SPECIFIED	174	HO	DANTRIUM, DANTROLENE	DEEP VEIN THROMBOSIS
144119171	14411917	20180119	NA	5	YR	NA	NA	M	OT	US	181	LT	DANTROLENE SODIUM	HEPATIC FAILURE
144123501	14412350	20180119	NA	5	YR	NA	NA	M	OT	US	181	OT	DANTROLENE SODIUM	CEREBRAL INFARCTION, COMPARTMENT SYNDROME, DISSEMINATED INTRAVASCULAR COAGULATION, HEPATIC FAILURE, PULMONARY HAEMORRHAGE, RESPIRATORY FAILURE, SHOCK
145883001	14588300	20180301	NA	5	YR	NA	NA	M	OT	US	181	HO	DANTROLENE SODIUM, SEVOFLURANE	LIVER INJURY
157310841	15731084	20181217	2018	NA	NA	NA	NA	NA	MD	US	184	OT	RYANODEX	BLINDNESS UNILATERAL, CHEST DISCOMFORT, HOT FLUSH, INCORRECT DRUG ADMINISTRATION RATE, INJECTION SITE EXTRAVASATION, MALAISE, RETINAL DETACHMENT, RETINAL TEAR
159023642	15902364	20190304	NA	36	YR	118	KG	M	MD	US	191	OT	DANTROLENE SODIUM, HYDROMORPHONE, PROPOFOL, MIDAZOLAM, LACTATED RINGERS SOLUTION, FENTANYL, ROCURONIUM, SUGAMMADEX	BRACHIOCEPHALIC VEIN THROMBOSIS

primaryid	CASEID	FDA_DT	EVENT_DT	AGE	AGE_COD	WT	WT_COD	SEX	OCCP_COD	REPORTER_COUNTRY	GetDataYear	OUTC_COD	DRUGNAME	PT
159334401	15933440	20190207	NA	36	YR	118	KG	M	MD	US	191	OT	DANTROLENE, MIDAZOLAM, LACTATED RINGERS SOLUTION, FENTANYL, PROPOFOL, ROCURONIUM, HYDROMORPHONE, SUGAMMADEX	DEEP VEIN THROMBOSIS POSTOPERATIVE
160326681	16032668	20190305	NA	36	YR	NA	NA	M	MD	US	191	OT	DANTROLENE SODIUM	DEEP VEIN THROMBOSIS
185105691	18510569	20201116	20201116	16	YR	NA	NA	M	PH	US	204	OT	DANTROLENE SODIUM, D5NS 20K 100 HR	PRODUCT DEPOSIT, PRODUCT PREPARATION ERROR, PRODUCT USE ISSUE
234874771	23487477	20240206	20180101	NA	NA	NA	NA	NA	CN	US	241	HO	DANTRIUM, SUCCINYLCHOLINE	INJECTION SITE EXTRAVASATION, AURA, RETINAL DETACHMENT, INTRAOCULAR PRESSURE INCREASED, RETINAL TEAR, BLINDNESS
4395241	4169876	20040706	NA	NA	NA	NA	NA	UNK	PH	NA	43	DE	DANTRIUM	MEDICATION ERROR
5465358	6436071	20070920	20070906	40	YR	78	KG	F	PH	UNITED STATES	73	HO	DANTROLENE SODIUM	BURNING SENSATION, CONTUSION, DRUG INTOLERANCE, ERYTHEMA, INFUSION RELATED, REACTION, OEDEMA PERIPHERAL, PAIN IN EXTREMITY, TENDERNESS
6465858	7211993	20091127	20090101	NA	NA	NA	NA	NA	MD	UNITED STATES	94	OT	DANTRIUM	NA
6677099	7378383	20100401	20090205	20	YR	NA	NA	M	PH	UNITED STATES	102	HO	DANTRIUM	INFUSION SITE THROMBOSIS
7646336	8069151	20110725	NA	32	YR	NA	NA	F	MD	UNITED STATES	113	OT	MANNITOL, DANTROLENE SODIUM, SUCCINYLCHOLINE CHLORIDE, LASIX, THIOPENTAL, THIOPENTAL SODIUM, LIDOCAINE, LIDOCAINE HYDROCHLORIDE, BICARBONATE SODIUM, BICARBONATE, FENTANYL, ISOFLURANE,NITROUS OXIDE W, OXYGEN, ATRACURIUM, ATRACURIUM BESILATE, NEOSTIGMINE	RESPIRATORY FAILURE

primaryid	CASEID	FDA_DT	EVENT_DT	AGE	AGE_COD	WT	WT_COD	SEX	OCCP_COD	REPORTER_COUNTRY	GetDataYear	OUTC_COD	DRUGNAME	PT
7646338	8069250	20110725	NA	50	YR	NA	NA	M	MD	UNITED STATES	113	OT	NITROUS OXIDE W, OXYGEN, CURARE ALKALOIDS, PROCAINAMIDE, DANTROLENE SODIUM, FENTANYL, THIOPENTAL, THIOPENTAL SODIUM, VECURONIUM BROMIDE, LASIX, SUCCINYLCHOLINE CHLORIDE, BICARBONATE, SODIUM BICARBONATE, ISOFLURANE	RESPIRATORY FAILURE
7646399	8069631	20110725	NA	44	YR	NA	NA	M	MD	UNITED STATES	113	OT	GLYCOPYRROLATE, ISOFLURANE, NEOSTIGMINE, LIDOCAINE, LIDOCAINE HYDROCHLORIDE, FENTANYL, BICARBONATE SODIUM, BICARBONATE, LABETALOL HCL, NITROUS OXIDE W OXYGEN, VECURONIUM BROMIDE, SUCCINYLCHOLINE CHLORIDE, PROPOFOL, INDERAL, DANTROLENE SODIUM, REGLAN, MIDAZOLAM HYDROCHLORIDE, ZOFRAN	INFUSION SITE EXTRAVASATION, OEDEMA PERIPHERAL
7646393	8069436	20110725	NA	41	YR	NA	NA	F	MD	UNITED STATES	113	OT	MIDAZOLAM HYDROCHLORIDE, BICARBONATE SODIUM BICARBONATE, NEOSTIGMINE NEOSTIGMINE, DANTROLENE SODIUM, GLYCOPYRROLATE, MANNITOL, SEVOFLURANE, ROCURONIUM BROMIDE, PROPOFOL, FENTANYL, NITROUS OXIDE W OXYGEN	RESPIRATORY FAILURE, SALIVARY HYPERSECRETION, CARDIAC DISORDER, DISSEMINATED INTRAVASCULAR COAGULATION, MUSCULAR WEAKNESS, PULMONARY OEDEMA
7646394	8069487	20110725	NA	69	YR	NA	NA	M	MD	UNITED STATES	113	OT	DIAZEPAM, BICARBONATE SODIUM BICARBONATE , PROCAINAMIDE, PROPOFOL, MANNITOL, BRETYLIUM BRETYLIUM , DANTROLENE SODIUM, PEPCID, LASIX, DESFLURANE, BUPIVACAINE HCL	RENAL IMPAIRMENT, PULMONARY OEDEMA, MUSCULAR WEAKNESS, CARDIAC DISORDER, HEPATIC FUNCTION ABNORMAL
7646396	8069546	20110725	NA	26	YR	NA	NA	M	MD	UNITED STATES	113	OT	ISOFLURANE, SUCCINYLCHOLINE CHLORIDE, BICARBONATE SODIUM, BICARBONATE, DANTROLENE SODIUM, LIDOCAINE LIDOCAINE HYDROCHLORIDE, ROCURONIUM BROMIDE, LASIX, PROPOFOL	PHLEBITIS, MUSCULAR WEAKNESS, DISSEMINATED INTRAVASCULAR COAGULATION, RENAL IMPAIRMENT, HEPATIC FUNCTION ABNORMAL, RESPIRATORY FAILURE
7646397	8069574	20110725	NA	31	YR	NA	NA	M	MD	UNITED STATES	113	OT	DANTROLENE SODIUM, ZOFRAN, ROCURONIUM BROMIDE, MIDAZOLAM HYDROCHLORIDE, LIDOCAINE LIDOCAINE, HYDROCHLORIDE, EPHEDRINE EPHEDRINE, HYDROCHLORIDE,	PHLEBITIS, RESPIRATORY FAILURE, RENAL IMPAIRMENT, DISSEMINATED

primaryid	CASEID	FDA_DT	EVENT_DT	AGE	AGE_COD	WT	WT_COD	SEX	OCCP_COD	REPORTER_COUNTRY	GetDataYear	OUTC_COD	DRUGNAME	PT
													DESFLURANE, MANNITOL, PROPOFOL, ATROPINE SULFATE, FENTANYL, LASIX, BICARBONATE SODIUM BICARBONATE	INTRAVASCULAR COAGULATION
7646399	8069631	20110725	NA	44	YR	NA	NA	M	MD	UNITED STATES	113	OT	GLYCOPYRROLATE, ISOFLURANE, NEOSTIGMINE NEOSTIGMINE, LIDOCAINE LIDOCAINE, HYDROCHLORIDE, FENTANYL, BICARBONATE SODIUM BICARBONATE, LABETALOL HCL, NITROUS OXIDE W OXYGEN, VECURONIUM BROMIDE, SUCCINYLCHOLINE CHLORIDE, PROPOFOL, INDERAL, DANTROLENE SODIUM, REGLAN, MIDAZOLAM HYDROCHLORIDE, ZOFRAN	INFUSION SITE EXTRAVASATION, OEDEMA PERIPHERAL
7646400	8069649	20110725	NA	34	YR	NA	NA	M	MD	UNITED STATES	113	OT	SUFENTANIL CITRATE, LASIX, PROPOFOL, NEOSTIGMINE NEOSTIGMINE, MIDAZOLAM HCL, ISOFLURANE, ROBINUL GLYCOPYRRROIUM, BROMIDE, NITROUS OXIDE W OXYGEN, SUCCINYLCHOLINE CHLORIDE, FENTANYL, DANTROLENE SODIUM, MANNITOL, VECURONIUM BROMIDE, LIDOCAINE LIDOCAINE, HYDROCHLORIDE	RESPIRATORY FAILURE, PHLEBITIS, PULMONARY OEDEMA, MUSCULAR WEAKNESS
7646427	8090201	20110725	NA	13	YR	NA	NA	F	MD	UNITED STATES	113	OT	DANTROLENE SODIUM	RESPIRATORY FAILURE, DEAFNESS TRANSITORY, MUSCULAR WEAKNESS
7646518	8068323	20110725	NA	17	YR	NA	NA	M	MD	UNITED STATES	113	OT	FENTANYL, DIAZEPAM, VECURONIUM BROMIDE, LIDOCAINE LIDOCAINE, HYDROCHLORIDE, DANTROLENE SODIUM, ISOFLURANE ISSOFLURANE, INSULIN, ZANTAC, DEXTROSE 5, BICARBONATE SODIUM BICARBONATE , THIOPENTAL THIOPENTAL SODIUM , SUCCINYLCHOLINE CHLORIDE	HYPERKALAEMIA
7646529	8068325	20110725	NA	10	YR	NA	NA	M	MD	UNITED STATES	113	OT	SOLU MEDROL, DEXTROSE 5, MANNITOL, BRETYLIUM BRETYLIUM, ZOFRAN, INSULIN, DANTROLENE SODIUM, BICARBONATE SODIUM, BICARBONATE, HALOTHANE, NITROUS OXIDE W OXYGEN	MUSCULAR WEAKNESS, RESPIRATORY FAILURE, ALTERED STATE OF CONSCIOUSNESS
7646532	8089449	20110725	NA	31	YR	NA	NA	M	MD	UNITED STATES	113	OT	GLYCOPYRROLATE, SEVOFLURANE, PROPOFOL, FENTANYL, NEOSTIGMINE NEOSTIGMINE, BICARBONATE SODIUM BICARBONATE, MIDAZOLAM HYDROCHLORIDE, SUCCINYLCHOLINE CHLORIDE, DANTROLENE SODIUM, ROCURONIUM BROMIDE, LASIX	RESPIRATORY FAILURE
7646533	8068335	20110725	NA	20	YR	NA	NA	M	MD	UNITED STATES	113	OT	DANTROLENE SODIUM, MORPHINE, DESFLURANE, ROCURONIUM BROMIDE, FENTANYL, NITROUS OXIDE W OXYGEN, MANNITOL, BICARBONATE SODIUM BICARBONATE, MIDAZOLAM MIDAZOLAM, HYDRCHLORIDE,	HYPERKALAEMIA, RESPIRATORY FAILURE, RENAL IMPAIRMENT

primaryid	CASEID	FDA_DT	EVENT_DT	AGE	AGE_COD	WT	WT_COD	SEX	OCCP_COD	REPORTER_COUNTRY	GetDataYear	OUTC_COD	DRUGNAME	PT
													SUCCINYLCHOLINE CHLORIDE, LASIX, DEXTROSE 5, INSULIN INSULIN	
7646536	8068357	20110725	NA	56	YR	NA	NA	M	MD	UNITED STATES	113	OT	EPINEPHRINE, GLYCOPYRROLATE, DEXTROSE 5, PROTAMINE PROTAMINE, HYDROCHLORIDE, CALCIUM CHLORIDE, PANCURONIUM PANCURONIUM BROMIDE, MANNITOL, SUFENTANIL CITRATE, NEOSTIGMINE NEOSTIGMINE, INSULIN INSULIN, AMICAR, FAMOTIDINE, BICARBONATE SODIUM BICARBONATE, MIDAZOLAM HYDROCHLORIDE, ETOMIDATE, SUCCINYLCHOLINE CHLORIDE, DANTROLENE SODIUM, PHENYLEPHRINE HCL, ISOFLURANE, LASIX	MUSCULAR WEAKNESS, CARDIAC OUTPUT DECREASED, HYPOTENSION
7646537	8068478	20110725	NA	58	YR	NA	NA	F	MD	UNITED STATES	113	OT	FAMOTIDINE, FENTANYL, THIOPENTAL THIOPENTAL SODIUM, INSULIN INSULIN, SUCCINYLCHOLINE CHLORIDE, LASIX, VECURONIUM BROMIDE, DANTROLENE SODIUM, DEXTROSE 5, ENFLURANE, BICARBONATE SODIUM BICARBONATE	HYPERKALAEMIA, CARDIAC DISORDER
7646541	8068484	20110725	NA	NA	YR	NA	NA	M	MD	UNITED STATES	113	OT	INSULIN, ATRACURIUM ATRACURIUM BESILATE, VECURONIUM BROMIDE, NTIROUS OXIDE NITROUS OXIDE, BICARBONATE SODIUM BICARBONATE, PROCAINAMIDE, ISOFLURANE, BRETYLIUM BRETYLIUM, DANTROLENE SODIUM, DEXTROSE 5, FENTANYL, THIOPENTAL THIOPENTAL SODIUM, MANNITOL	HYPERKALAEMIA, MUSCULAR WEAKNESS, CEREBROVASCULAR ACCIDENT
7646544	8068491	20110725	NA	8	YR	NA	NA	M	MD	UNITED STATES	113	OT	HALOTHANE, BICARBONATE SODIUM BICARBONATE, ENFLURANE, NITROUS OXIDE W OXYGEN, DANTROLENE SODIUM, LIDOCAINE LIDOCAINE, HYDROCHLORIDE	RESPIRATORY FAILURE, MUSCULAR WEAKNESS
7646557	8068527	20110725	NA	60	YR	NA	NA	M	MD	UNITED STATES	113	OT	DANTROLENE SODIUM, FENTANYL, LIDOCAINE LIDOCAINE, HYDROCHLORIDE, MANNITOL, PANCURONIUM PANCURONIUM, BICARBONATE SODIUM BICARBONATE, PHENYLEPHRINE HCL, MIDAZOLAM HYDROCHLORIDE, ISOFLURANE, LASIX	RESPIRATORY FAILURE, MUSCULAR WEAKNESS
7646560	8068529	20110725	NA	50	YR	NA	NA	M	MD	UNITED STATES	113	OT	DEXTROSE 5, ISOFLURANE, DOPAMINE HCL, LASIX, VECURONIIUM VECURONIUM, NEOSTIGMINE NEOSTIGMINE, METILSULFATE, EPHEDRINE EPHEDRINE, HYDROCHLORIDE, BICARBONATE SODIUM BICARBONATE, INSULIN, DANTROLENE SODIUM, GLYCOPYRROLATE, PENTOBARBITAL CAP, ATIVAN LORAZAPEM, FENTANYL, MANNITOL, THIOPENTAL THIOPENTAL SODIUM, LEVOPHED NOREPINEPHRINE BITRATE	CARDIAC OUTPUT DECREASED
7646836	8089832	20110725	NA	6	YR	NA	NA	M	MD	UNITED STATES	113	OT	NITROUS OXIDE W OXYGEN, LIDOCAINE LIDOCAINE, HYDROCHLORIDE, DANTROLENE SODIUM, ISOFLURANE, LASIX, HALOTHANE	RESPIRATORY FAILURE
7646859	8069019	20110725	NA	23	YR	NA	NA	M	MD	UNITED STATES	113	DE	DANTROLENE SODIUM, INSULIN, ATROPINE SULFATE, DEXTROSE 5, CALCIUM CHLORIDE, ISOFLURANE, LASIX, LEVOPHED NOREPINEPHRINE BITARTRATE VASOPRESSOR, PROPOFOL, DESFLURANE, MIDAZOLAM HYDROCHLORIDE, ROCURONIUM BROMIDE, ANCEF, DILAUDID, BICARBONATE SODIUM BICARBONATE, VASOPRESSOR, FENTANYL	HYPOTENSION, HYPERKALAEMIA, CARDIAC DISORDER
7646868	8070328	20110725	NA	49	YR	NA	NA	M	MD	UNITED STATES	113	OT	SUCCINYLCHOLINE CHLORIDE, FENTANYL, DESFLURANE, ZOFRAN, MIDAZOLAM HYDROCHLORIDE, DEXTROSE 5, REGLAN, CALCIUM CHLORIDE, MANNITOL,	PULMONARY OEDEMA, DISSEMINATED INTRAVASCULAR

primaryid	CASEID	FDA_DT	EVENT_DT	AGE	AGE_COD	WT	WT_COD	SEX	OCCP_COD	REPORTER_COUNTRY	GetDataYear	OUTC_COD	DRUGNAME	PT
													ESMOLOL ESMOLOL, PROPOFOL, DOPAMINE HCL, DANTROLENE SODIUM, ROCURONIUM BROMIDE, LASIX, INSULIN, BICARBONATE SODIUM BICARBONATE, KAYEXALATE	COAGULATION, MUSCULAR WEAKNESS, PHLEBITIS, RESPIRATORY FAILURE, EYE DISORDER
8605118	8684454	20120807	NA	18	YR	NA	NA	M	OT	UNITED STATES	123	DE	DANTROLENE SODIUM, SUCCINYLCHOLINE CHLORIDE, SEVOFLURANE	CARDIAC ARREST, CARDIAC FAILURE, PULMONARY OEDEMA, COAGULOPATHY, HAEMORRHAGE, VENTRICULAR, ARRHYTHMIA, HYPERTHERMIA MALIGNANT, BLOODY DISCHARGE, PUNCTURE SITE HAEMORRHAGE, ORGAN FAILURE
92245292	9224529	20130924	NA	NA	NA	NA	NA	M	OT	US	133	OT	DANTROLENE	BLOOD CREATINE PHOSPHOKINASE INCREASED, HYPERTHERMIA MALIGNANT
99101341	9910134	20140212	NA	41	YR	NA	NA	F	OT	US	141	DE	DANTROLENE, BROMOCRIPTINE BROMOCRIPTINE, PROPOFOL, ZIPRASIDONE, LITHIUM, LAMOTRIGINE, CLONAZEPAM, BENZTROPINE, CHLOMIPRAMINE	NA

sTable4. case by case of JADER database in malignant hyperthermia patients.

primaryid	number	sex	age	wt	ht	file	progress	report_source	occp_cod	drugname	PT
AB-06026059	2	F	10			200604	调查完了	自发报告	医师;药剂师	ダントロレンナトリウム水和物, ダントリウム, ニカルジピン塩酸塩, ドパミン塩酸塩, デクスメデトミジン塩酸塩, プロポフォール	ARRHYTHMIA, CARDIAC FAILURE
AB-06026061	2	F	10			200604	调查完了	自发报告	医师;药剂师	ニカルジピン塩酸塩, ペルジピン, ダントロレンナトリウム水和物, ドパミン塩酸塩, デクスメデトミジン塩酸塩, プロポフォール	ARRHYTHMIA, CARDIAC FAILURE
AB-23068451	1	M	50			202304	调查完了	自发报告	药剂师	ダントロレンナトリウム水和物, ダントリウム, ニトログリセリン, ニカルジピン, セファゾリン, グリセリン, プロポフォール, フロセミド	ASPARTATE AMINOTRANSFERASE INCREASED

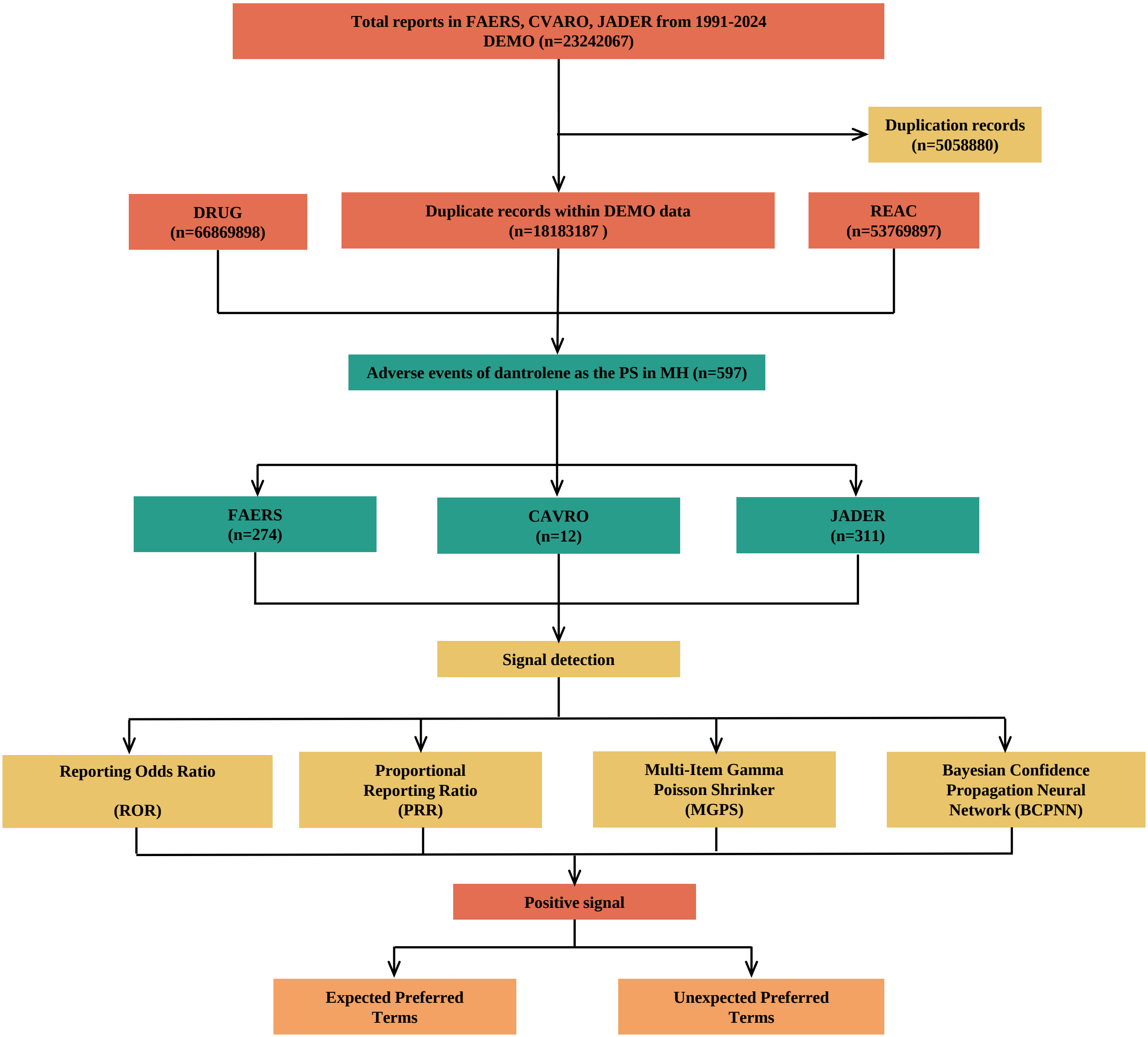

Total reports in FAERS, CVARO, JADER from 1991-2024
DEMO (n=23242067)
Duplication records
(n=5058880)
DRUG
(n=66869898)
Duplicate records within DEMO data
(n=18183187)
REAC
(n=53769897)
Adverse events of dantrolene as the PS in MH (n=597)
FAERS
(n=274)
CAVRO
(n=12)
JADER
(n=311)
Signal detection
Reporting Odds Ratio
(ROR)
Proportional
Reporting Ratio
(PRR)
Multi-Item Gamma
Poisson Shrinker
(MGPS)
Bayesian Confidence
Propagation Neural
Network (BCPNN)
Positive signal
Expected Preferred
Terms
Unexpected Preferred
Terms
